# Somatic Copy Number Alterations in Human Cancers: An Analysis of Publicly Available Data From The Cancer Genome Atlas

**DOI:** 10.3389/fonc.2021.700568

**Published:** 2021-07-28

**Authors:** Luuk Harbers, Federico Agostini, Marcin Nicos, Dimitri Poddighe, Magda Bienko, Nicola Crosetto

**Affiliations:** ^1^Division of Genome Biology, Department of Medical Biochemistry and Biophysics, Karolinska Institutet, Stockholm, Sweden; ^2^Bienko-Crosetto Lab, Science for Life Laboratory, Stockholm, Sweden; ^3^Department of Pneumonology, Oncology and Allergology, Medical University of Lublin, Lublin, Poland; ^4^Department of Medicine, Nazarbayev University School of Medicine, Nur-Sultan, Kazakhstan; ^5^Clinical Academic Department of Pediatrics, National Research Center for Maternal and Child Health, University Medical Center, Nur-Sultan, Kazakhstan

**Keywords:** copy number alterations, cancer, TCGA, cosmic genes, 3D genome

## Abstract

Somatic copy number alterations (SCNAs) are a pervasive trait of human cancers that contributes to tumorigenesis by affecting the dosage of multiple genes at the same time. In the past decade, The Cancer Genome Atlas (TCGA) and the International Cancer Genome Consortium (ICGC) initiatives have generated and made publicly available SCNA genomic profiles from thousands of tumor samples across multiple cancer types. Here, we present a comprehensive analysis of 853,218 SCNAs across 10,729 tumor samples belonging to 32 cancer types using TCGA data. We then discuss current models for how SCNAs likely arise during carcinogenesis and how genomic SCNA profiles can inform clinical practice. Lastly, we highlight open questions in the field of cancer-associated SCNAs.

## Introduction

### A Bit of Semantics

Cancer cells almost invariably harbor altered karyotypes. Deviations from the normal karyotype can range from entire or partial gains or losses of chromosomes and large intra-chromosomal inversions or translocations between different chromosomes, to more complex rearrangements, such as “chromothripsis” (where massive genomic rearrangements are generated in a single event and are localized to isolated chromosomal regions) (1). Inversions, translocations, and complex rearrangements are usually referred to as structural variants (SVs). On the other hand, there is considerable confusion in the literature as to the terminology used to describe different deviations from the normal copy number state of diploid cells (2*n*) (2). Strictly speaking, any deviation from the 2*n* copy number state of a region in the genome represents a copy number alteration (CNA). However, gains or losses of entire chromosomes have historically been referred to as aneuploidy or whole chromosome aneuploidy, while gains or losses of chromosomal arms or smaller chromosomal regions have been variably termed segmental or partial aneuploidy, (focal) CNAs or small insertions or deletions (indels), depending on the size of the genomic region amplified or deleted (2). The mechanisms responsible for whole chromosome CNAs, arm-level CNAs, focal CNAs and indels are different—as we discuss below—therefore calling for the use of separate terms. However, for simplicity, we adopt the convention recently proposed by Ben-David and Amon (2) and use: (i) the term aneuploidy to describe all CNAs affecting either entire chromosome arms (excluding the short arms of acrocentric chromosomes) or whole chromosomes; (ii) the term CNAs to describe all sub-arm gains or losses larger than 10 kilobases (kb); and (iii) the term indels to describe all other CNAs. Specifically, here we refer to CNAs arising post-zygotically in a somatic cell as somatic CNAs or SCNAs. In contrast, we refer to CNAs that occur in the germline, and are therefore inheritable, as copy number variants or CNVs.

### The Landscape of SCNAs Across Human Cancers

Since the launch of The Cancer Genome Atlas (TCGA) and International Cancer Genome Consortium (ICGC) initiatives in 2005 and 2008, respectively, SCNAs have been profiled in thousands of tumor samples in virtually all cancer types using high density single nucleotide polymorphism (SNP) arrays. Recently, the Pan-Cancer Analysis of Whole Genomes (PCAWG) consortium of TCGA and ICGC further expanded the already huge repertoire of publicly available cancer SCNA datasets, by profiling more than 2,600 cancers by whole genome sequencing (1, 3). A summary of the TCGA and PCAWG studies in which SCNAs have been profiled and/or analyzed is available as **Supplementary Table 1**. All these datasets are derived from primary tumors. Recently, the first pan-cancer whole genome analysis of metastatic tumors became available, which includes high resolution SCNA profiles from 2,520 samples (4). Altogether, these data offer a unique opportunity to explore the prevalence, type, and genomic distribution of SCNAs in different tumor types. In turn, this information can be used to guide hypotheses about the mechanisms of formation and evolution of SCNAs during tumorigenesis.

The first comprehensive pan-cancer analysis of SCNAs was published in 2013 and was based on a still relatively small set (*n* = 4,934) of TCGA samples, which were available at that time (5). This study revealed broad differences in the prevalence of SCNAs across different tumor types, with some cancers such as ovarian carcinomas having a large fraction of their genome either amplified or deleted (5). The same study was also the first to reveal recurrent patterns of focal amplifications and deletions involving known cancer genes, such as amplifications of *CCND1*, *EGFR*, *MYC* and deletions of *CDKN2A*, *CDKN2B* and *STK11* (5). Since then, most pan-cancer analyses have focused on mutations in cancer genes and on mutational patterns, and only a few of them have specifically focused on SCNAs. In one of these analyses (6), 39,568 SCNAs from 3,131 tumors sequenced by TCGA were integrated with genome wide maps of chromosome contact frequencies measured with high-throughput chromosome conformation capture (Hi-C) (7). This analysis was the first to suggest a link between the genomic landscape of cancer SCNAs and the three-dimensional (3D) architecture of the genome, and to report a different distribution of amplifications and deletions within the 3D genome (6). More recently, the association between the 3D genome and cancer SCNAs was further investigated by the PCAWG consortium, by integrating whole genome SV profiles from 2,658 cancers representing 38 tumor types with available Hi-C data from multiple cell lines (8). This comprehensive analysis revealed that deletions are more frequently found within so-called topologically associating domains (TADs) (9) and lamina associated domains (LADs) (10)—two types of structural domains shaping the 3D genome—whereas duplications tend to span multiple TADs and are more frequently detected within inter-LAD regions, suggesting that these alterations arise through different mechanisms. Integration of SCNA and TAD annotations, together with gene expression profiles, has also been used to identify the presence of rearrangements involving *cis*-regulatory elements, such as enhancers, within cancer genomes (11). This approach led to the discovery of enhancer hijacking events that result in the activation of the *IRS4* gene in a subset of lung cancers and of the *IGF2* gene in a subset of colorectal cancers, by bringing these genes in physical proximity to active enhancers that are normally not contacted by the same genes (11).

Although very insightful, we argue that pan-cancer analyses such as those mentioned above, which intersect various omics data with databases of SCNAs, need to be placed in the right context by a pan-cancer analysis of the SCNAs themselves, including a survey of their type, prevalence, and distribution across the genome. An analysis of this sort was recently performed on the prevalence and type of aneuploidies across 10,249 tumors and 32 cancer types using TCGA data (12), but it did not consider SCNAs as we define them here. In this article, we therefore re-analyze all the available SCNA datasets from TCGA and assess their prevalence, type and genomic distribution across different tumor types, and which cancer associated genes are mostly affected by these SCNAs. We then discuss the (limited) available information about how SCNAs likely emerge and evolve during the process of tumorigenesis and highlight open research questions in the field of cancer-associated SCNAs. We acknowledge that TCGA data do not provide an exhaustive portrait of genomic alterations in cancers and that important differences exist between the TCGA cohort and the U.S. general population of cancer patients (13), not to mention the rest of the world. We therefore warn the Reader against generalizing the conclusions of our analysis outside of the TCGA population.

## Methods

### TCGA Datasets

We downloaded all the TCGA data and associated clinical information by creating a Genomic Data Commons (GDC) manifest of primary tumors using the R (version 4.0.3) package *GenomicDataCommons* (version 1.12.0) (14). We then filtered the manifest based on primary tumors for which copy number data are available. To obtain the relevant clinical data we translated TCGA barcodes using the R package *TCGAutils* (15) (version 1.8.1).

### Analysis of SCNA Frequency Across Different Tumor Types

We called genomic regions with a log2 ratio above 0.32 as amplified, whereas we called genomic regions with a log2 ratio below –0.42 as deleted. We then classified amplified and deleted genomic regions as following: (i) indels if they were smaller than 10 kb in size; (ii) aneuploidies if they spanned more than 75% of a chromosomal arm; (iii) SCNAs for all the other alterations. We computed the total percentage of the genome that was either amplified or deleted and the frequency of each alteration for every tumor sample, separately for all tumor types. When computing the alteration frequency per chromosome, we normalized the values by chromosome length. Finally, we computed the P values for the total percentage and for the total number of amplifications and deletions for each tumor type using the R function *wilcox.test()* and the Spearman correlation using the R function *cor()*, which are both available in the *stats* package (version 4.0.3).

### Analysis of SCNA Lengths

We computed the average length of SCNAs for every tumor sample, separately for all tumor types. We also calculated the average SCNA length for each chromosome, with and without normalization by chromosome length. We calculated the P values for the mean SCNA length of amplifications and deletions using the R function *wilcox.test()* which is available in the *stats* package (version 4.0.3).

### Analysis of COSMIC Genes Affected by SCNAs

To generate a list of genes associated with different tumor types, we downloaded the COSMIC Cancer Gene Census (16), a list of 723 cancer associated genes. Following this, we overlapped the genomic regions of these 723 genes with the previously called SCNAs regions in all the TCGA samples analyzed. Genes are called amplified or deleted if there is at least a 50% overlap of the gene with a SCNA.

### Analysis of Co-Amplified and Co-Deleted COSMIC Genes

To generate the list of genes pairs that are simultaneously affected by SCNAs, we analyzed all pair combinations of the 723 COSMIC genes and reported the frequency of all the possible co-occurring events (*i.e.*, AMP-AMP, DEL-DEL and AMP-DEL) across all experiments for each tumor type (see **Supplementary Table 2**).

## Results

We analyzed the prevalence, type, and genomic distribution of 853,218 SCNAs across 10,729 tumor samples belonging to 32 cancer types (see *Methods*), being guided by the following five key questions.

### How Much of the Genome Is Affected by SCNAs in Different Cancer Types?

**Figure 1:**
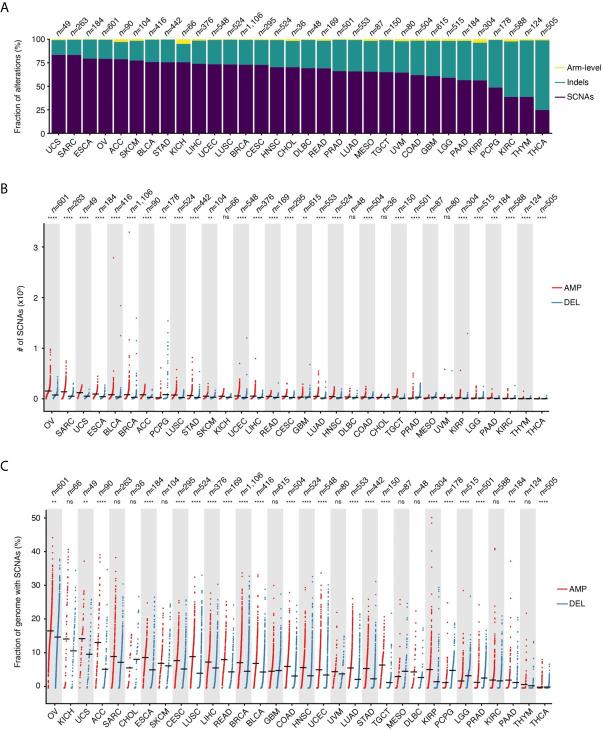
Prevalence of SCNAs across different tumor types. **(A)** Percentage of arm-level aneuploidies, indels and SCNAs by tumor type. **(B)** Number of SCNAs by tumor type, separately for amplifications (AMP) and deletions (DEL). Each dot represents one tumor sample. Black horizontal bars: mean values. **(C)** Same as in **(B)** but considering the percentage of the reference genome either amplified or deleted in each tumor. *n*, number of samples analyzed for each tumor type. In **(B, C)**, asterisks indicate statistical significance levels (Wilcoxon’s test, two-sided) at 0.05 (*), 0.01 (**), 0.001 (***) and 0.0001 (****). ns, not statistically significant. See **Supplementary Table 4** for the list of tumor type abbreviations used.

SCNAs defined as amplifications or deletions longer than 10 kb and spanning at most 75% of the corresponding chromosome arm represent the main type of alteration in 28 out of 32 (87.5%) tumor types for which copy number data are available in TCGA (**Figure 1A**). Indels represent the second most frequent type of alteration in these tumors, while they prevail over SCNAs in thyroid carcinomas (THCA), thymomas (THYM), kidney renal clear cell carcinomas (KIRC), and pheochromocytomas and paragangliomas (PCPG). SCNAs account for more than 80% of all copy number changes detected in uterine corpus endometrial carcinomas (UCEC) and in sarcomas (SARC). When examining the total number of SCNAs and the percentage of the genome affected by them, ovarian carcinomas (OV) and SARC carry, on average, the highest burden of SCNAs, followed by uterine carcinosarcoma (UCS), esophageal carcinomas (ESCA), bladder urothelial carcinomas (BLCA), and breast invasive carcinomas (BRCA) (**Figures 1B, C**). There is considerable heterogeneity in the number of SCNA events per sample within the same tumor type, with OV, SARC, BRCA, and PCPG showing the highest variability (**Figure 1B**). Similarly, the percentage of the genome altered varies extensively from tumor to tumor within the same type, with some tumors having more than 30% of the genome either amplified or deleted (**Figure 1C**). In most cancer types, amplifications are significantly more abundant and affect a larger fraction of the genome compared to deletions (**Figures 1B, C**). However, in the same tumor type, the number of amplifications is, on average, correlated with the number of deletions (**Supplementary Figure 1A**). In multiple tumor types, the number of amplifications and deletions is also significantly correlated within the same sample (**Supplementary Figure 1B**), suggesting that in these tumors both SCNA types are driven by common mechanism(s).

### What Is the Average Size of SCNAs?

**Figure 2:**
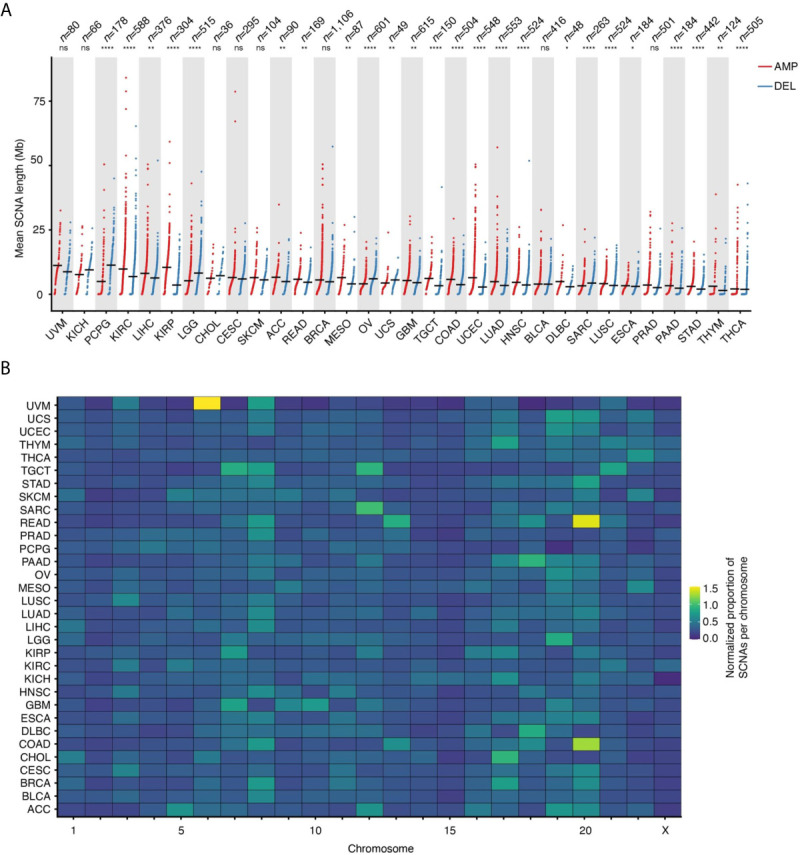
Length and proportion of SCNAs across chromosomes. **(A)** Length of SCNAs by tumor type, separately for amplifications (AMP) and deletions (DEL). Each dot represents the average alteration length in one tumor sample. Black horizontal bars: mean values. Asterisks indicate statistical significance levels (Wilcoxon’s test, two-sided) at 0.05 (*), 0.01 (**), 0.001 (***) and 0.0001 (****). ns, not statistically significant. **(B)** Proportion of SCNAs in each chromosome normalized by chromosome length for each of the 32 tumor types analyzed. The normalization was done as following: (# of SCNAs in a chromosome)/(tot # of SCNAs) * (chromosome length in Mb). See **Supplementary Table 4** for the list of tumor type abbreviations used.

Across the 32 tumor types examined, the mean SCNA length is 3.83 ± 7.89 megabases (Mb, mean ± s.d.), with deletions spanning longer distances on average (mean length: 4.69 Mb *vs.* 3.38 Mb for deletions and amplifications, respectively). The trend, however, is highly dependent on the tumor type (**Figure 2A**). Some chromosomes tend to harbor longer SCNAs: for example, chromosome (chr) 13 and 15 have the longest SCNAs in uveal melanomas (UVM), whereas chr1 and chr2 carry the longest SCNAs in KIRC and kidney renal papillary cell carcinomas (KIRP) (**Supplementary Figure 2A**). Interestingly, after normalizing by chromosome length, the group including chr13–22 shows proportionally larger SCNAs compared to chr1–12 and chrX (**Supplementary Figure 2B**). Although the basis of these differences in SCNA length is unknown, it might be related to variable chromatin composition and localization of these two groups of chromosomes within the cell nucleus in different cell types.

### Which Chromosomes Are Most Frequently Affected by SCNAs?

In addition to certain chromosomes harboring longer or shorter SCNAs, there is also a clear propensity for SCNAs to occur within certain chromosomes, even when accounting for differences in chromosome length. For instance, a relatively large proportion of SCNA events are found within chr8 across many tumor types, whereas most of the SCNAs detected in adenocarcinomas of the colon (COAD) and rectum (READ) fall within chr13 and chr20, and most of the SCNAs in BRCA and cholangiocarcinomas (CHOL) affect chr17 while other chromosomes, such as chr15, are largely unaffected by SCNAs (**Figure 2B**). Notably, chr8 harbors the *MYC* gene, which is one of the most frequently amplified genes in human cancers (17), whereas chr17 contains the *ERBB2* gene, which is amplified in about 20% of all invasive breast cancers (18). However, the fact that some chromosomes or chromosomal regions appear to be more susceptible to SCNAs in human cancers might be independent of the fitness advantage imparted by the amplification or deletion of specific genes. For example, this could depend on differences in the amount of DNA damage and type or efficiency of DNA repair along individual chromosomes. Indeed, using methods that probe the frequency of endogenous DNA double-strand breaks (DSBs) at high resolution along the genome, we and others have demonstrated that these lesions—which likely underpin the formation of many SCNAs—form non-randomly along the genome as a result of the interplay between DNA replication, transcription and 3D genome dynamics (19–25).

### Which Cancer Genes Are Most Frequently Amplified or Deleted?

**Figure 3:**
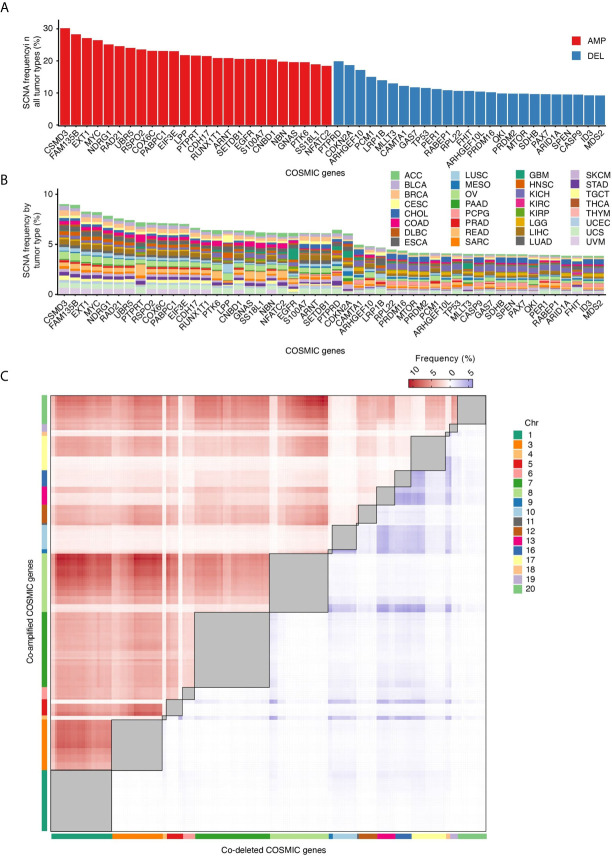
COSMIC genes most frequently affected by SCNAs. **(A)** Percentage of all tumor types in which the 25 most frequently amplified (red) and deleted (blue) COSMIC genes are altered. **(B)** Percentage of tumors in which each COSMIC gene shown in **(A)** is altered in each of the 32 tumor types analyzed. **(C)** Frequency of COSMIC gene pairs that are either co-amplified (red) or co-deleted (blue) in at least 5% of all the 10,729 tumors analyzed. Intra-chromosomal pairs, which are the majority, are masked (grey squares) to make the inter-chromosomal pairs visible. The full list of inter-chromosomal pairs (including AMP-DEL events) sorted by tumor type is available in **Supplementary Table 2**.

In tumors, SCNAs are thought to confer a fitness advantage to the cells that harbor them, by leading to increased levels of expression of certain genes and decreased expression of others (26). We therefore examined which among the genes listed in the Catalogue Of Somatic Mutations in Cancer (COSMIC) (16) are most frequently altered by SCNAs. *CSMD3* and *FAM135B*, which encode proteins of unknown function, are the first and second most frequently amplified genes, whereas *PTBRD* and *CDKN2A*, which encode a receptor tyrosine phosphatase and a cyclin-dependent kinase inhibitor, respectively, are the first and second most frequently deleted genes across all the 10,729 tumors that we examined (**Figure 3A**). Some genes share similar SCNA frequency spectra, which in some cases depends on the fact that these genes are localized near each other along the linear genome (**Figure 3B**). Other genes have unique SCNA frequency spectra and are proportionally more frequently altered in certain tumor types (**Figure 3B**). For example, the *PTPRT* and *PTK6* genes, which encode a receptor tyrosine phosphatase and a cytoplasmic tyrosine kinase, respectively, are proportionally more frequently amplified in COAD and READ. *EGFR*, which encodes a receptor tyrosine kinase, is more frequently amplified in glioblastomas, whereas *LRP1B*, which encodes a low-density lipoprotein receptor related protein, is predominantly deleted in lung adenocarcinomas (LUAD). These differences likely reflect the relative functional importance of these genes in different tissues, and, as a result, the fitness advantage imparted by their dysregulation in different tumor types.

### Are Certain Cancer Genes Frequently Co-Amplified or Co-Deleted?

SCNAs may affect multiple cancer genes at the same time because of their proximity along the linear genome. Indeed, COSMIC gene pairs mapped to the same chromosome arm display a much higher frequency of co-amplifications (AMP-AMP) and co-deletions (DEL-DEL) compared to genes located on different chromosomes (**Supplementary Figure 3**). But what about pairs of cancer genes located on different chromosomes? When we analyzed the frequency of AMP-AMP and DEL-DEL events involving COSMIC gene pairs located on different chromosomes, several interesting features emerged (**Figure 3C**). Firstly, inter-chromosomal co-amplifications occur at substantially higher frequency compared to co-deletions, similar to what we observed for paired intra-chromosomal SCNAs. This agrees with the long-standing observation that chromosome gains are far better tolerated than chromosome losses (26). Secondly, the frequency of AMP-AMP and DEL-DEL events is not evenly distributed across all chromosomes: chr1, chr3, chr7 and chr8 account for most of the visible clusters of co-amplifications, whereas co-deletions predominantly involve cancer genes located on chr13, chr16, chr17 and chr18. Thirdly, a small number of cancer genes accounts for most co-amplification or co-deletion events. The *MYC* gene is involved in 31 out of the 100 most frequent AMP-AMP pairs across different chromosomes in the 10,729 tumors analyzed, followed by *FAM135B* (19%) and *NDRG1* (15%) (**Supplementary Tables 2, 3**). Among the top 100 DEL-DEL pairs, *ARHGEF10* is engaged in 31% of the co-deletion events, followed by *PCM1* (20%) and *CDKN2A* (13%) (**Supplementary Tables 2, 3**). These preferred co-amplification and co-deletion events likely depend on both the effect of the selection forces acting during tumor evolution—which might favor certain SCNA combinations over others—as well as on the genomic context in which SCNAs arise. For example, the fact that AMP-AMP are more frequent on larger chromosomes and DEL-DEL on smaller ones might be related to differences in the radial placement and/or frequency of reciprocal contacts between these chromosomes inside the cell nucleus.

## Discussion

Our analysis of publicly available TCGA data clearly shows that SCNAs are pervasive across human cancers and that certain tumor types have a considerable portion of their genome affected by SCNAs. What remains unclear is how cancer associated SCNAs form in the first place, and how (if) they evolve during tumor progression. The molecular mechanisms of SCNA formation have been extensively studied in yeast and reviewed elsewhere (27). In contrast, very little is known about how SCNAs arise in mammalian cells and during tumorigenesis. This is not surprising, considering that the etiology of SCNAs in tumors remains unclear and it is very challenging to recapitulate them experimentally. In the following paragraphs we discuss the available scientific evidence that informs our current view of how SCNAs might form and evolve during tumorigenesis, by distinguishing three main sources of evidence: 1) integrative genomic analyses; 2) *in vitro* experiments; and 3) phylogenetic analyses based on multi-region or single-cell sequencing of pre-malignant lesions, primary tumors, and metastases. Importantly, the mechanisms of SCNA formation are different from those that cause aneuploidy, which have been reviewed elsewhere (2, 12) and will not be discussed here.

### Integrative Genomic Analyses

Integrative analyses of SCNAs and (epi)genomic features associated with DNA fragility and damage can suggest potential mechanisms by which SCNAs arise during tumorigenesis or help formulate hypotheses that can be tested experimentally. By intersecting genome-wide DSB maps with SCNA data available from the early stage of TCGA, we previously showed that cancer-associated deletions and, to a lesser extent, amplifications significantly overlap with genomic regions where DSBs accumulate upon exposure of cells to mild doses of the DNA polymerase inhibitor aphidicolin—a treatment that causes replication stress (19). Subsequently, a pan-cancer analysis intersected somatic focal (≤ 5 Mb) deletions identified by TCGA in 10,221 tumor specimens spanning 30 cancer types with known common fragile sites (CFSs), to assess whether deletions occur more frequently at these sites than elsewhere in the genome (28). CFSs are megabase-sized genomic regions that tend to break frequently upon induction of replication stress and are cytogenetically detected as chromosome gaps or breaks in metaphase spread preparations (28). This analysis revealed that CFSs significantly overlap with the boundaries of focal deletions detected in cancer, suggesting that replication stress might represent the driving force of (at least some of) these alterations in human cancers (28). Although insightful, these analyses only provide correlative evidence and cannot pinpoint the exact causes and mechanisms of SCNA formation in cancer.

### *In Vitro* Experiments

One way to experimentally investigate the etiopathogenesis of SCNAs would be to expose normal or cancer cells to a variety of DNA damaging agents or genetic perturbations that favor the accumulation of DNA damage, followed by monitoring the emergence of SCNAs at the single-cell level at different timepoints. This approach has long been hindered by the lack of suitable methods for detecting SCNAs in single cells. However, in the past decade, several groups have begun exploring the formation of SCNAs *in vitro* using single-cell readouts. As mentioned above, replication stress causes the accumulation of DSBs at common fragile sites, which are enriched for breakpoints of cancer-associated deletions (28). Prompted by this observation, pioneering experiments based on single-cell cloning and array comparative genomic hybridization (aCGH) showed that acute exposure of cells to DNA replication stressors, such as aphidicolin or hydroxyurea, results in rapid formation of SCNAs at CFSs *in vitro* (29, 30). Analysis of breakpoint junctions revealed that these SCNAs were characterized by short (< 6 bp) microhomologies, suggesting that they formed through non-homologous end-joining (NHEJ) or another replication-coupled process, such as template switching (30). Subsequent integration of maps of SCNAs induced *in vitro* with gene expression profiles from the same cells revealed that long (> 1 Mb) transcribed genes overlapping with CFSs represent hotspots of SCNA formation following replication stress (31). More recently, the development of single-cell whole genome sequencing (WGS) methods has enabled more explorations of the emergence of SCNAs upon exposure of cells to DNA damaging agents *in vitro.* By applying single-cell WGS to diploid cells exposed to aphidicolin throughout one cell division cycle, different types of replication stress-induced SCNAs were identified, including a group of large (> 20 Mb) SCNAs associated with late DNA replication timing, low expression of nearby genes, and proximity to large genes, as well as a group of short (≤ 20 Mb) amplifications with contrasting associations with various genomic features (32). Recently, another single-cell genome sequencing method named direct nuclear tagmentation and RNA sequencing (DNTR-seq) was used to profile SCNAs induced by exposing HT116 colon carcinoma cells to high doses of the topoisomerase II inhibitor, etoposide, or to ionizing radiation *in vitro* (33). This study revealed that single copy deletions were the most common type of SCNAs caused by etoposide treatment and ionizing radiation, and that the probability of a copy number change within a given genomic region increased with the distance of the region from the centromere (33). Unfortunately, the same study did not compare the landscape of these SCNAs with those emerging following replication stress. Therefore, it is not possible at this point to conclude whether different types of DNA damage result in different genomic landscapes of SCNAs, and whether the type of cell in which the damage occurs influences the type and genomic distribution of the SCNAs formed.

### Phylogenetic Analyses

In addition to understanding the causes and mechanisms of SCNA formation in tumors, a fundamental question is when SCNAs arise in tumorigenesis and if/how the genomic landscape of SCNAs changes during tumor evolution. The SCNAs that are detected in a tumor at any given timepoint during the history of the disease represent the result of the continuous interplay between the mutagenic processes that trigger their formation (genome instability) and the selection forces (either positive or negative) that act upon them based on their impact on cellular fitness. A glimpse into these complex evolutionary dynamics is offered by comparative genomic studies that have analyzed SCNAs in the same tumor type at different disease stages. Comparison of precursor lesions and invasive melanomas revealed that SCNAs were prevalent only in the latter (34), suggesting that SCNAs are not an early DNA damage event during melanoma formation. In contrast, multi-region SCNA profiling in biopsies of Barret’s esophagus—the precursor lesion of esophageal adenocarcinoma—revealed the presence of multiple clonal SCNAs already at this early stage (35). Notably, the SCNA formation rate inferred using a Bayesian phylogenetic analysis was low in these precursor lesions (approx. 0.005 events per year per locus per allele), suggesting that, once they have been acquired in a rapid ‘burst’, SCNAs remain relatively stable throughout tumor evolution (35). This ‘punctuated evolution’ model has also been used to explain the evolution of SCNAs in triple-negative breast cancers (36), colorectal cancers (37) and pancreatic cancers (38). On the other hand, a recent single-cell WGS study conducted on 8 triple-negative breast cancers and 4 cell lines concluded that SCNAs continue to form during the evolution of these tumors, even though the majority of SCNAs are acquired in a single ‘burst’ in the early stages of tumorigenesis (39). Continuous SCNA evolution has also been suggested by numerous studies comparing primary (P) and metastatic (M) tumors or different regions within P and M lesions. For example, a comparison of P and M samples from patients with castration resistant prostate cancer found that the SCNA burden was significantly higher in M compared to P samples (40). Another study, in which SCNAs were compared between paired P-M samples from 10 patients with advanced prostate cancer, revealed that the SCNA landscape likely continued to evolve both in the primary tumor and in the metastases after dissemination (41). Variable P-M divergence in SCNA patterns has also been reported for colon (42–44), breast (45–48), lung (49) and renal cancers (50). Similarly, numerous studies based on multi-region tumor sequencing have consistently shown that the genomic pattern of SCNAs often differs between spatially distinct regions of the same tumor (51–54). In line with this, using a novel method that allows profiling SCNAs in multiple regions of individual formalin-fixed paraffin embedded tumor sections, we were able to show that SCNA genomic profiles can vary substantially between different regions of matched P-M breast cancer samples (55). Collectively, the results of these studies indicate that SCNAs likely originate in various ways in different tumor types and suggest that the genomic landscape of SCNAs is rather dynamic throughout tumor evolution, presumably due to the continuous interplay between genome instability and the selection forces at play. In this context, it is worth mentioning that many genes involved in the DNA damage response (DDR) and different types of DNA damage repair are frequently mutated in human cancers (56, 57), which likely contributes to fuel genomic instability and SCNA accumulation during tumor progression.

## Conclusion

It is by now clear that SCNAs constitute a pervasive and distinctive trait of human cancers, which carries independent prognostic information and likely reflects the level and type of genomic instability characteristic of each tumor. However, even though SCNAs have been profiled across thousands of tumor samples, we still know remarkably little about the mechanisms and processes that lead to their emergence during tumorigenesis, and how (if) dynamic these alterations are in tumor cells. This is mainly because, for ethical and practical reasons, it is not possible to take multiple biopsies of the same tumor at different stages of its growth—which would allow monitoring how SCNAs form and evolve *in vivo*. However, the availability of tractable *in vitro* systems, such as tumor organoids (58), alongside with single-cell genome sequencing methods (59) and CRISPR technologies for genome editing (60) should enable such investigations in the near future. We propose that frequent sampling and single-cell genome sequencing could be used to trace the formation and evolution of SCNAs in cells grown for prolonged periods of time in the presence of genetic or environmental cues that might cause or facilitate the formation of SCNAs (*e.g.*, DNA replication or transcription stressors). Patient-derived tumor xenografts (PDX) (61) represent another powerful tool for studying if and how the genomic landscape of SCNAs changes during the course of tumor evolution. Indeed, such models are already providing precious insights into the evolutionary dynamics of actionable and resistance-conferring point mutations (62).

A largely unexplored question is how SCNAs affect the spatial arrangement of the genome and whether this contributes to changes in gene expression and ultimately cell fitness. It is plausible that large amplifications or deletions, as well as other types of genomic rearrangements, will result in repositioning of genes and regulatory regions in their immediate neighborhood or even in changes to the overall 3D architecture of the genome. For example, using a novel method that we recently developed to infer radial genomic positions in the cell nucleus, we were able to show that translocations lead to substantial changes in the radial placement of the two parts of the same chromosome which get separated by the translocation, that in turn leads to large groups of consecutive genes being placed in either more active or more repressed chromatin neighborhoods (63). In the future, it will be important to develop methods that can probe SCNAs together with the 3D genome conformation and gene expression in single cells, to test their interplay and gain a deeper understanding of the mechanisms by which SCNAs convey a fitness advantage to the tumor cells that carry them.

Finally, SCNAs might represent an unexplored ‘Achille's heel’ of tumors, which could serve as a point of attack for novel therapeutic strategies. We hypothesize that, in cells with highly rearranged genomes and a high burden of SCNAs, the 3D genome might be in an unstable equilibrium, which would render them vulnerable to pharmacological perturbation of factors responsible for maintaining a certain arrangement of chromatin in 3D. Additionally, cells carrying many SCNAs might be more susceptible to disruption of key DNA repair pathways or to further accumulation of SCNAs, for example by treatment with DNA replication and/or transcription stressors. Exploring these possibilities will likely open new therapeutic avenues and ideally lead to more effective treatments for metastatic cancers, which, tragically, remain largely incurable.

## Data Availability Statement

The original contributions presented in the study are included in the article/**Supplementary Material**. Further inquiries can be directed to the corresponding author.

## Author Contributions

*Conceptualization*, NC. *Literature summary*, MN, DP. *TCGA data analysis*, LH, FA. *Funding acquisition*, NC, MB. *Supervision*, NC. *Visualization*, LH, FA, MB, NC. *Writing*, NC with contributions from all the authors. All authors contributed to the article and approved the submitted version.

## Funding

This work was supported by funding from the H2020-MSCA-ITN-2018 Marie Sklodowska-Curie Actions Innovative Training Networks (‘aDDRess’, grant no. 812829) to LH; the Polish National Science Center (grant no. UMO- 2016/23/D/NZ2/02890) to MN; the Swedish Cancer Research Foundation (grant no. 19 0130 Pj 03 H) to MB; and the Swedish Cancer Research Foundation (grant no. CAN 2018/728), the Strategic Research Programme in Cancer (StratCan) at Karolinska Institutet (grant no. 2201) and the Swedish Foundation for Strategic Research (grant no. BD15_0095) to NC.

## Conflict of Interest

The authors declare that the research was conducted in the absence of any commercial or financial relationships that could be construed as a potential conflict of interest.

## Publisher’s Note

All claims expressed in this article are solely those of the authors and do not necessarily represent those of their affiliated organizations, or those of the publisher, the editors and the reviewers. Any product that may be evaluated in this article, or claim that may be made by its manufacturer, is not guaranteed or endorsed by the publisher.
