# Supplementary material for: Somatic Copy Number Alterations in Human Cancers: An Analysis of Publicly Available Data From The Cancer Genome Atlas
Source: Front Oncol. 2021 Jul 28;11:700568. doi: 10.3389/fonc.2021.700568 (PMC8355892; doi:10.3389/fonc.2021.700568)
Supplement: Supplementary file 1 [file DataSheet_1.docx]

Supplementary Material

**Somatic copy number alterations in human cancers:**

**prevalence, mechanisms, and clinical implications**

**Luuk Harbers, Federico Agostini, Marcin Nicos, Dimitri Poddighe,**

**Magda Bienko & Nicola Crosetto**

1. Supplementary Figures pg. 2
2. Supplementary Tables pg. 6

## 1. Supplementary Figures


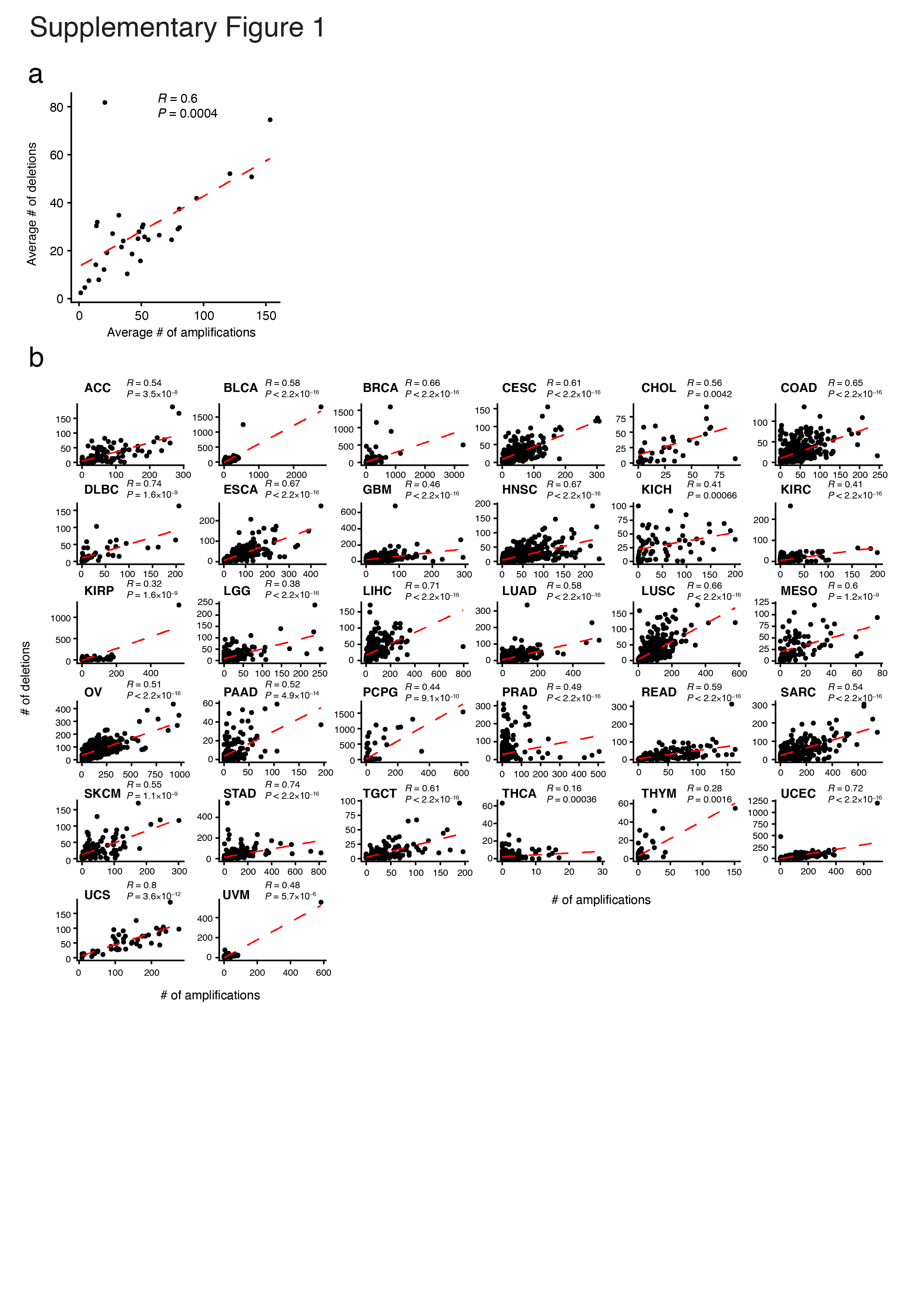


**Supplementary Fig. 1.** (**a**) Correlation between the average number of amplification and deletion events in the 32 tumor types analyzed. Each dot represents one tumor type. (**b**) Correlation between the number of amplifications and deletions in each of the 10,729 tumors analyzed. Each dot represents one tumor sample. In both (a) and (b), Spearman’s correlation (R) and the associated P-values (p) are shown, and the red dashed line represents the linear regression fit. The tumor type acronyms used in (b) are listed in **Supplementary Table 2**.


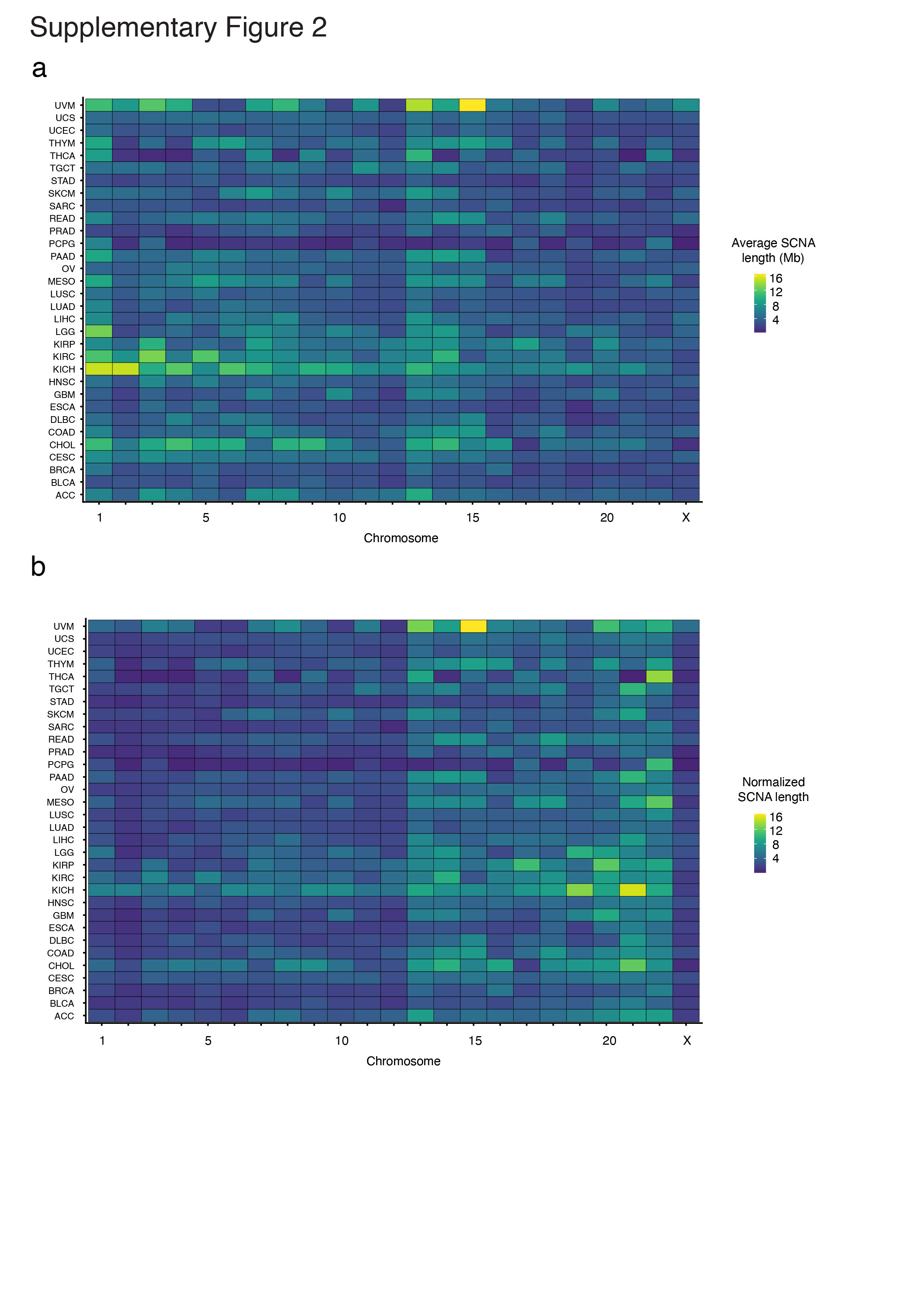


**Supplementary Fig. 2.** (**a**) Average SCNA length by chromosome for each of the 32 tumor types analyzed. Amplifications and deletions are considered together. (**b**) Same as in (a) but after normalization by chromosome length. The tumor type acronyms are listed in **Supplementary Table 2**.


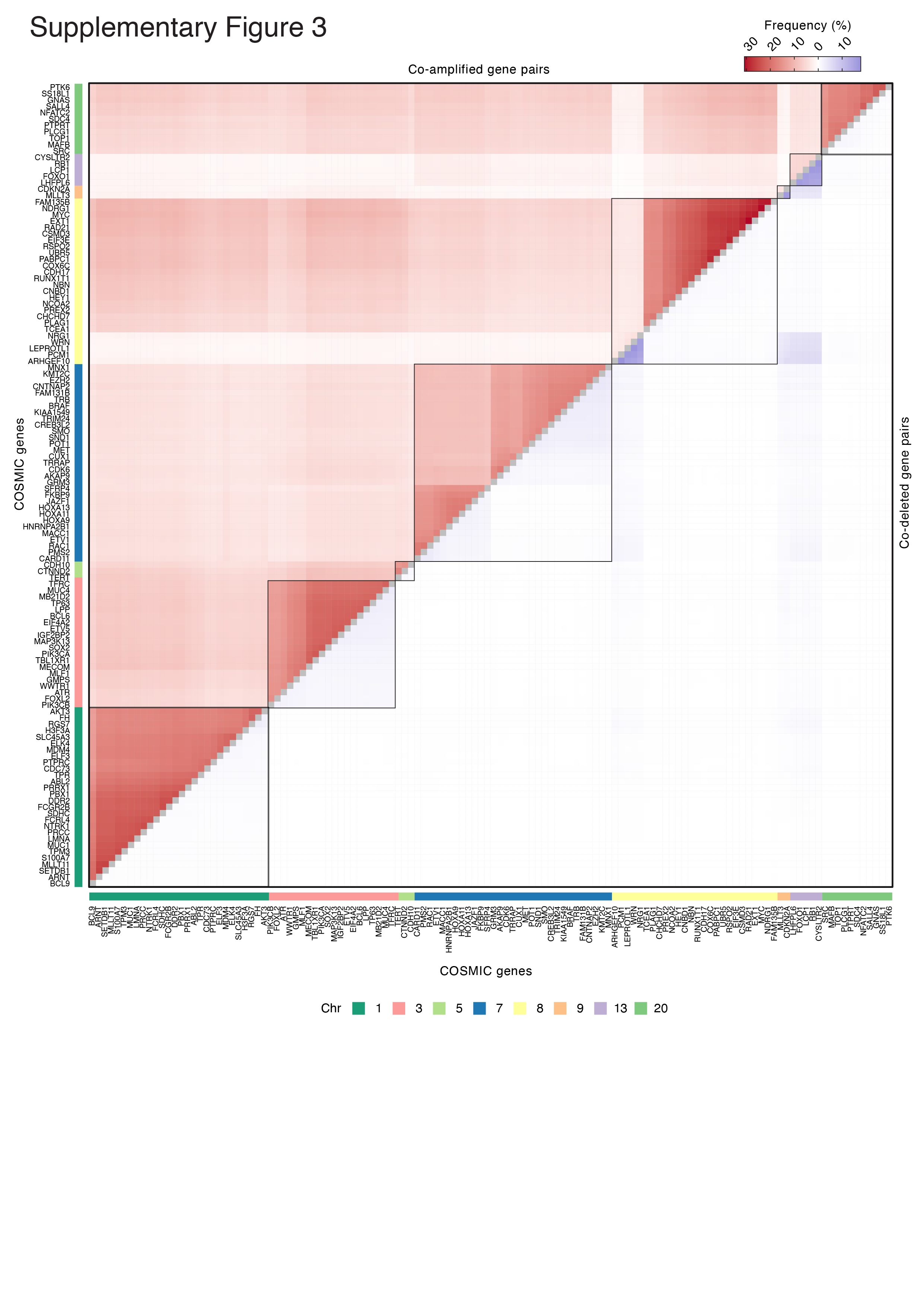


**Supplementary Fig. 3.** Frequency of COSMIC gene pairs that are either co-amplified (red) or co-deleted (blue) in at least 15% of the 10,729 tumors analyzed.

**2. Supplementary Tables**

**Supplementary Table 1.** Summary of TCGA and PCAWG papers that report SCNAs in different cancer types.

| **PMID** | **Year** | **Consortium** | **Cancer type*** | **# Samples** | **Method** | **Resolution** | **Caller** |
| --- | --- | --- | --- | --- | --- | --- | --- |
| 18772890 | 2008 | TCGA | GBM | 91 | microarray | 25kb | GISTIC |
| 21720365 | 2011 | TCGA | OV | 316 | microarray | 25kb | BLAT |
| 22810696 | 2012 | TCGA | COAD, READ | 97 | WGS | NA | NA |
| 23000897 | 2012 | TCGA | BRCA | 773 | microarray | 25kb | GISTIC |
| 22960745 | 2012 | TCGA | LUSC | 178 | microarray | 25kb | GISTIC |
| 23636398 | 2013 | TCGA | UCEC | 365 | microarray | 25kb | GISTIC |
| 23634996 | 2013 | TCGA | LAML | 200 | microarray | 35kb | DNACopy |
| 23792563 | 2013 | TCGA | KIRC | 417 | microarray | 25kb | GISTIC |
| 24120142 | 2013 | TCGA | GBM | 543 | microarray | 25kb | GISTIC |
| 24476821 | 2014 | TCGA | BLCA | 131 | WGS, microarray | 25kb | GISTIC |
| 25079552 | 2014 | TCGA | LUAD | 230 | WGS, microarray | 25kb | GISTIC |
| 25109877 | 2014 | TCGA | Pan-cancer | 3527 | microarray | NA | NA |
| 25079317 | 2014 | TCGA | GIAC | 295 | microarray | 25kb | GISTIC |
| 25417114 | 2014 | TCGA | PTC | 495 | microarray | 25kb | GISTIC |
| 25631445 | 2015 | TCGA | HNSC | 279 | WGS, microarray | 25kb | GISTIC |
| 26091043 | 2015 | TCGA | SKCMC | 333 | microarray | 25kb | GISTIC |
| 26061751 | 2015 | TCGA | LGG | 285 | microarray | 25kb | GISTIC |
| 26451490 | 2015 | TCGA | BRCA | 817 | microarray | 25kb | GISTIC |
| 26544944 | 2015 | TCGA | PRAD | 333 | microarray | 25kb | GISTIC |
| 26536169 | 2016 | TCGA | KIRP | 161 | microarray | 25kb | GISTIC |
| 27165744 | 2016 | TCGA | ACC | 89 | microarray | 25kb | GISTIC |
| 27158780 | 2016 | TCGA | LUSC, LUAD | 1114 | microarray | 25kb | GISTIC |
| 28052061 | 2017 | TCGA | ESCA | 164 | microarray | 25kb | GISTIC |
| 28112728 | 2017 | TCGA | CESC | 228 | microarray | 25kb | GISTIC |
| 28622513 | 2017 | TCGA | LIHC | 363 | microarray | 25kb | GISTIC |
| 28810144 | 2017 | TCGA | PAAD | 150 | microarray, WES | focal | GISTIC |
| 29438696 | 2017 | TCGA | THYM | 117 | microarray | 25kb | GISTIC |
| 29617669 | 2018 | TCGA | KIRC | 843 | microarray | 25kb | GISTIC |
| 29625053 | 2018 | TCGA | Pan-cancer | NA | microarray | NA | NA |
| 29622463 | 2018 | TCGA | Pan-cancer | 10522 | microarray | 25kb | GISTIC |
| 29622464 | 2018 | TCGA | OV, UCEC, CESC, UCS, BRCA | 2579 | microarray | 25kb | GISTIC |
| 29622466 | 2018 | TCGA | GIAC | 921 | microarray | 25kb | GISTIC |
| 29489755 | 2018 | PCAWG | Pan-cancer | 1699 | WGS | 10kb | ACEseq |
| 32025012 | 2020 | PCAWG | Pan-cancer | 2658 | WGS | 10kb | ACEseq |
| 32025015 | 2020 | PCAWG | Pan-cancer | 2658 | WGS | 20kb | GISTIC |
| 32025003 | 2020 | PCAWG | Pan-cancer | 2658 | WGS | 20kb | GISTIC |

*See Supplementary Table 2 below for the specification of acronyms.

**Supplementary Table 2.** Co-amplification or co-deletion frequencies of COSMIC gene pairs located on different chromosomes, separately for each of the 32 tumor types analyzed. Because of its large size, this table is provided as a separate Excel file.

**Supplementary Table 3.** 100 most frequently co-amplified or co-deleted COSMIC gene pairs located on different chromosomes across the 10,729 tumors analyzed. Because of its large size, this table is provided as a separate Excel file.

**Supplementary Table 4.** Tumor type abbreviations adopted by TCGA and used in this article.

| **TCGA acronym** | **Tumor type** |
| --- | --- |
| LAML | Acute Myeloid Leukemia |
| ACC | Adrenocortical carcinoma |
| BLCA | Bladder Urothelial Carcinoma |
| LGG | Brain Lower Grade Glioma |
| BRCA | Breast invasive carcinoma |
| CESC | Cervical squamous cell carcinoma and endocervical adenocarcinoma |
| CHOL | Cholangiocarcinoma |
| LCML | Chronic Myelogenous Leukemia |
| COAD | Colon adenocarcinoma |
| CNTL | Controls |
| ESCA | Esophageal carcinoma |
| FPPP | FFPE Pilot Phase II |
| GBM | Glioblastoma multiforme |
| GIAC | Gastrointestinal tract adenocarcinoma |
| HNSC | Head and Neck squamous cell carcinoma |
| KICH | Kidney Chromophobe |
| KIRC | Kidney renal clear cell carcinoma |
| KIRP | Kidney renal papillary cell carcinoma |
| LIHC | Liver hepatocellular carcinoma |
| LUAD | Lung adenocarcinoma |
| LUSC | Lung squamous cell carcinoma |
| DLBC | Lymphoid Neoplasm Diffuse Large B-cell Lymphoma |
| MESO | Mesothelioma |
| MISC | Miscellaneous |
| OV | Ovarian serous cystadenocarcinoma |
| PAAD | Pancreatic adenocarcinoma |
| PCPG | Pheochromocytoma and Paraganglioma |
| PRAD | Prostate adenocarcinoma |
| PTC | Papillary thyroid carcinoma |
| READ | Rectum adenocarcinoma |
| SARC | Sarcoma |
| SKCM | Skin Cutaneous Melanoma |
| STAD | Stomach adenocarcinoma |
| TGCT | Testicular Germ Cell Tumors |
| THYM | Thymoma |
| THCA | Thyroid carcinoma |
| UCS | Uterine Carcinosarcoma |
| UCEC | Uterine Corpus Endometrial Carcinoma |
| UVM | Uveal Melanoma |
